# Management of ischaemic stroke survivors in primary care setting: the road to holistic care

**DOI:** 10.1007/s11739-023-03445-y

**Published:** 2023-10-24

**Authors:** Hizir Ozdemir, Dimitrios Sagris, Azmil Husin Abdul-Rahim, Gregory Yoke Hong Lip, Eduard Shantsila

**Affiliations:** 1 Liverpool Centre for Cardiovascular Science at University of Liverpool, Liverpool John Moores University and Liverpool Heart & Chest Hospital, Liverpool, UK; 2 Department of Internal Medicine, School of Health Sciences, Faculty of Medicine, University of Thessaly, Larissa, Greece; 3 Department of Cardiovascular and Metabolic Medicine, Institute of Life Course and Medical Sciences, Faculty of Health and Life Sciences, University of Liverpool, Liverpool, UK; 4 Department of Clinical Medicine, Aalborg University, Aalborg, Denmark; 5 Department of Primary Care and Mental Health, University of Liverpool, Liverpool, UK

**Keywords:** Stroke, Atrial fibrillation, Anticoagulation, Primary care

## Abstract

The management of ischaemic stroke survivors is multidisciplinary, necessitating the collaboration of numerous medical professionals and rehabilitation specialists. However, due to the lack of comprehensive and holistic follow-up, their post-discharge management may be suboptimal. Achieving this holistic, patient-centred follow-up requires coordination and interaction of subspecialties, which general practitioners can provide as the first point of contact in healthcare systems. This approach can improve the management of stroke survivors by preventing recurrent stroke through an integrated post-stroke care, including appropriate **A**ntithrombotic therapy, assisting them to have a **B**etter functional and physiological status, early recognition and intervention of **C**omorbidities, and lifestyles. For such work to succeed, close interdisciplinary collaboration between primary care physicians and other medical specialists is required in a holistic or integrated way.

## Introduction

Ischaemic stroke is a major cause of long-term disability worldwide [1]. Advance in the medical management of stroke reduced stroke mortality and increased the number of people discharged home after a stroke [2]. For stroke survivors and their families, hospital discharge is just the beginning of a long journey. Many stroke survivors experience recurrent cerebrovascular events or complications within months or years of their initial event [3]. Management of complications and prevention of recurrent stroke necessitates a multidisciplinary approach from various healthcare providers, including stroke specialists, general practitioners, cardiologists, internists, radiologists, speech and rehabilitation specialists, and psychologists. All these experts should share goals and objectives following a common post-stroke integrated care plan to reduce cardiovascular morbidity and mortality.

Due to a lack of holistic follow-up, stroke survivors and their caregivers may feel abandoned. Since general practitioners (GPs) are the first point of contact with the healthcare system in many countries, they are in an ideal position to coordinate subspecialties to optimise chronic disease management, including complications and comorbidities. They play an important role in secondary prevention after stroke by controlling clinical risk factors and optimising overall management.

**Fig. 1:**
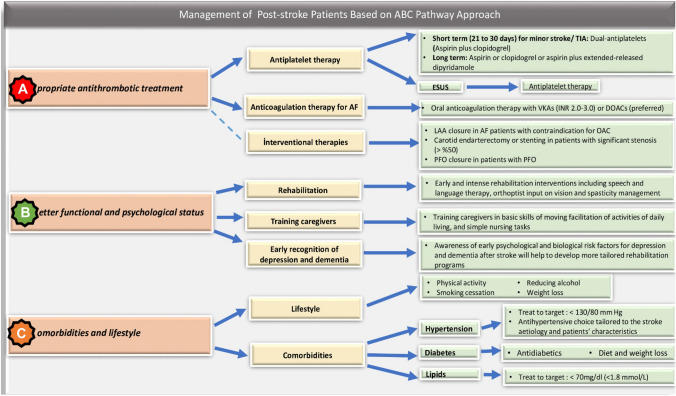
Management of post-stroke patients based on ABC pathway approach. *TIA*  transients ischemic attack, *ESUS* embolic stroke of undetermined source, *VKA* vitamin-K antagonists, *INR* international normalised ratio, *DOAC* direct-acting oral anticoagulant, *LAA* left atrial appendage, *AF*  atrial fibrillation, *OAC* oral anticoagulant, *PFO* patent foramen ovale

Multiple pieces of evidence and speciality guidelines guide primary care physicians in the complex management of people with a previous stroke. This review summarises this literature and highlights strategies for preventing recurrent stroke, maximising function, and identifying and managing complications. This broadly follows the post-stroke ABC pathway approach, recently recommended in a position paper from the European Society of Cardiology Council on Stroke [4]: **A** for *Appropriate antithrombotic therapy*, **B** for *Better functional and psychological status,* and **C** for *Comorbidities and lifestyle, patient values and preferences (*Fig. 1*).*

A move to an integrated care pathway approach has been advocated for many long-term conditions [5]. In patients with atrial fibrillation (AF), for example, adherence to an integrated care pathway has been associated with improved clinical outcomes, leading to its recommendation in international guidelines. [6, 7]

## [A]ppropriate antithrombotic therapy

Antithrombotic therapy is the backbone of secondary stroke prevention based on the underlying potential stroke aetiology and the individualised indications or contraindications. It is an essential component of an integrated care pathway for stroke management.

### Antiplatelet therapy

Antiplatelet therapy is the standard antithrombotic strategy in most patients with non-cardioembolic ischaemic strokes. Current guidelines suggest long-term antithrombotic therapy with a single antiplatelet agent, either low-dose aspirin or clopidogrel, as standard [8, 9]. The combination of aspirin and extended-release dipyridamole is still recommended in secondary stroke prevention by current AHA/ASA (American Heart Association/American Stroke Association) guidelines [8]. However, the Royal College of Physicians’ guidelines recommends a combination of aspirin and dipyridamole only in patients who cannot tolerate clopidogrel.

However, this combination may be more difficult to tolerate, with a higher discontinuation rate than clopidogrel [10]. Ticagrelor is a new potent P2Y12 inhibitor. Although it may have a role in secondary stroke prevention, especially in patients with large artery intracranial atherosclerosis and after recurrent events [11] the evidence of its effectiveness and safety is limited and should be initiated by secondary care [8]. The role of cilostazol in patients with a previous stroke as monotherapy or in addition to aspirin or clopidogrel has been investigated in RCTs, providing promising results, but these studies only aimed Asian population, thus limiting their generalizability [12, 13].

Although long-term dual antiplatelet with aspirin and clopidogrel is contraindicated in patients with ischaemic stroke due to the increased risk of major bleeding [14], in patients with minor stroke (National Institutes of Health Stroke Scale [NIHSS] ≤ 3) or a high-risk transient ischaemic attack (ABCD2 ≥ 4), the use of dual-antiplatelet therapy for 21 days is effective and safe [15, 16]. After the first 3-week period, the risk of major bleeding significantly increases. Thus the use of dual antiplatelets for up to 90 days is not universally recommended [15]. The THALES study in patients with mild stroke (NIHSS ≤ 5) or high-risk TIA (ABCD2 ≥ 6) showed that the combination of ticagrelor and aspirin had a lower risk of composite stroke or death within 30 days than aspirin monotherapy at the cost of increased risk of severe bleeding [17]. Subgroup analysis of the THALES study revealed that the combination therapy was only beneficial in patients with 30% or greater large artery stenosis, which was not associated with an increased risk of bleeding [11]. The CHANCE-2 trial demonstrated that compared to clopidogrel plus aspirin, the combination of ticagrelor and aspirin modestly lowered the risk of stroke at 90 days in patients with minor ischaemic stroke or TIA who were carriers of CYP2C19 loss-of-function alleles, but with more bleeding events [18]. Given the limitations, initiation of such treatments should be initiated by secondary care for selected patients.

Despite an extensive diagnostic work-up for acute ischaemic stroke, the cause of stroke remains unexplained in 20% of patients (i.e. cryptogenic stroke) [19]. The term embolic stroke of undetermined source (ESUS) was introduced to reclassify these patients identifying those who may benefit more from oral anticoagulation [19]. Two large RCTs, the NAVIGATE ESUS (Rivaroxaban Versus Aspirin in Secondary Prevention of Stroke and Prevention of Systemic Embolism in Patients With Recent Embolic Stroke of Undetermined Source) and RE-SPECT ESUS (Dabigatran Etexilate for Secondary Stroke Prevention in Patients With Embolic Stroke of Undetermined Source) showed that anticoagulation with rivaroxaban and dabigatran was not superior to aspirin for secondary stroke prevention in unstratified patients with ESUS [20, 21]. Thus, patients who fall into the category of ESUS should be treated with antiplatelet therapy.

Patients with patent foramen ovale (PFO) are at risk of stroke due to venous thromboembolism. PFO-related stroke is more common in people with migraines. Patients with PFO should be treated with PFO closure and/or antiplatelet therapy since anticoagulation is neither more effective nor safer than antiplatelets [22].

A recurrent stroke may occur despite the recommended antithrombotic therapy. Such patients should be investigated for other stroke causes, especially to identify occult AF [23]. If AF or other cause suggesting the use of anticoagulation is not present, patients with recurrent stroke can be switched to another antiplatelet regimen [24]. Ensuring good treatment adherence is particularly important in such cases. Even though short atrial high rate episodes may significantly increase the risk of stroke [25], it is unclear whether stroke patients with short atrial runs would benefit from using oral anticoagulants to reduce their risk of stroke [26]. The increasing use of cardiac implantable devices, external monitors and smart watches provides an opportunity for prolonged rhythm to assess the AF burden for stroke survivors and optimise antithrombotic treatment. The importance of detection of AF is evident, especially when even asymptomatic AF episodes carry a poor prognosis, and more prolonged or sophisticated monitoring approaches can help improve the detection of AF [27].

### Anticoagulation therapy

Oral anticoagulation (OAC) is the treatment of choice for the prevention of thromboembolic events and especially ischaemic stroke. OACs include vitamin-K anticoagulants (VKA) and non-vitamin-K oral anticoagulants (NOAC), which are as effective as and safer than VKAs [28]. In patients with previous ischaemic stroke, NOACs significantly reduce the risk of stroke or systemic embolism, haemorrhagic stroke, and major bleeding compared to VKAs [29].

Stroke survivors with AF and stable atherosclerotic disease (i.e. coronary artery disease, carotid disease, peripheral artery disease) should receive OAC monotherapy [30]. Recently, among 2236 patients with stable coronary artery disease, rivaroxaban monotherapy was non-inferior to a combination of rivaroxaban and antiplatelet treatment for the composite outcome of stroke, systemic embolism, myocardial infarction, unstable angina requiring revascularization, or death from any cause (HR 0.72; 95% CI 0.55 to 0.95; *P* < 0.001 for noninferiority). Also, it was associated with a lower risk of major bleeding (HR 0.59, 95% CI 0.39 to 0.89; *P* = 0.01 for superiority) [31]. Combination therapy can be considered in patients with AF presenting with acute coronary syndromes, especially when undergoing coronary stenting, when it may reduce the possibility of stent thrombosis [32]. In these patients, the duration of triple antithrombotic therapy may vary from 1 to 6 months, balancing the risk of stent thrombosis and bleeding, with dual antithrombotic typically continued for no more than 1 year and followed by OAC alone [32].

Apart from AF, anticoagulation therapy in secondary stroke prevention may be considered with caution in other situations. In patients with a mechanical valve, irrespective of AF, dabigatran was associated with increased thromboembolic risk and bleeding complications compared with warfarin [33]. Thus, NOACs are contraindicated in patients with mechanical valves. Recent data suggest that in patients with AF and a bioprosthetic mitral valve placed more than 3 months previously, rivaroxaban was non-inferior to VKA [34]. These data point towards the use of NOACs also in patients with bioprosthetic valves.

Finally, it is common for patients with AF to have concomitant atherosclerotic disease related to multiple cardiovascular risk factors [35]. The presence of high-grade carotid stenosis in patients with stroke has therapeutic implications for other aspects of secondary prevention such as high-intensity lipid-lowering treatment or carotid revascularisation interventions [36, 37].

Overall, the selection of different antiplatelet and anticoagulant therapies for preventing secondary stroke relies on the stroke aetiology, bleeding risk profile, compliance, tolerance to the treatment and potentially, drug resistance (defined as treatment failure or by laboratory tests). Before commencing an antiplatelet/anticoagulant treatment doctors should carefully consider the relative benefits and bleeding risks for each patient. Further research is needed to clarify optimal duration of dual-antiplatelet therapy in patients with symptomatic intracranial atherosclerosis since the benefits are primarily seen short-term while the bleeding risk is high throughout the prolonged therapy. Currently, there is inadequate evidence to advocate genetic testing as a standard of care and RCTs are required to assess CYP2C19 gene testing-based antiplatelet therapy for stroke prevention.

## [B]etter functional and psychological status

Improvements in the medical management of stroke patients led to a considerable reduction in post-stroke morbidity and mortality. Despite the expanding interventions in secondary stroke prevention, many stroke patients suffer from significant disabilities, resulting in impaired quality of life [2]. Early recognition of patients who benefit more from intense rehabilitation programmes may result in better functional outcomes [38]. Therefore, to provide holistic post-stroke care and achieve the best functional recovery in stroke patients, an integrated care system must incorporate a network between stroke physicians, GPs and rehabilitation teams. Several medical societies provide recommendations to guide post-stroke rehabilitation interventions, including speech and language therapy by early community stroke team and orthoptist input on vision and spasticity management; however, these recommendations may vary according to guidelines and countries [39]. Therefore, they should be tailored to each patient.

Due to moderate to severe disability, stroke survivors need assistance, and family caregivers are the key to taking care of stroke patients. Primary care plays a vital role in the long-term care of stroke survivors, with effective follow-up and information supply and facilitating transfer back to specialist services if required. Training caregivers in basic skills of moving and handling, facilitation of activities of daily living, and simple nursing tasks reduces the burden of care and improves the quality of life of patients and caregivers [40]. Caregiver training has the additional advantages of reducing the costs of stroke care and improving patients’ quality of life. Also, it helps patients to gain independence at an earlier stage [40]. However, due to a lack of holistic follow-up, stroke survivors and their caregivers may feel abandoned.

Depression is a major cause of loss of independence after stroke; within a year after stroke, a third of survivors experience post-stroke depression [41]. This affects their active participation in rehabilitation and treatment adherence and results in poor quality of life and higher mortality [42]. Dementia is another common post-stroke complication with a similar prevalence of approximately 30% among stroke survivors and is also one of the leading causes of dependency [43]. Cognitive impairment may co-exist with depression, especially in elderly stroke survivors. One randomised trial showed that early recognition and successful treatment of the depressive disorder improved post-stroke impairment of cognitive function [44]. Awareness of early psychological and biological risk factors for depression and dementia after stroke will help to develop more tailored rehabilitation programmes, including psychological interventions. In both situations, awareness and a multidisciplinary approach are required, with neuropsychological testing tailored to the clinical situation. Effective partnerships with patients, their families, and carers help recognise early symptoms of depression and dementia, assist primary care doctors in timely treatments, and connect with broader post-stroke services for early interventions.

Finally, post-stroke care requires a well-organised comprehensive approach, including early and intensive rehabilitation, caregiver training, and early recognition of depression and dementia. Current medical practise is often focused on inpatient rehabilitation and better holistic transitional and community rehabilitation is needed following the transition from acute phase to long-term rehabilitation [45–47]. Furthermore, insufficient long-term community and outpatient rehabilitation resources contribute to unmet needs during long-term rehabilitation and care for post-stroke patients. Further improvements are required to establish a standardised rehabilitation delivery system and to develop projects to promote long-term rehabilitation. The provision of adequate therapy for post-stroke patients necessitates active board-certified physiatrist involvement; however, due to a lack of board-certified physiatrists. Additionally, many medical schools lack a department dedicated to teaching rehabilitation medicine. Establishing such a department would help students to learn about medical care and comprehensive post-stroke rehabilitation.

## [C]omorbidities and lifestyle, patient values, and preferences

**Fig. 2:**
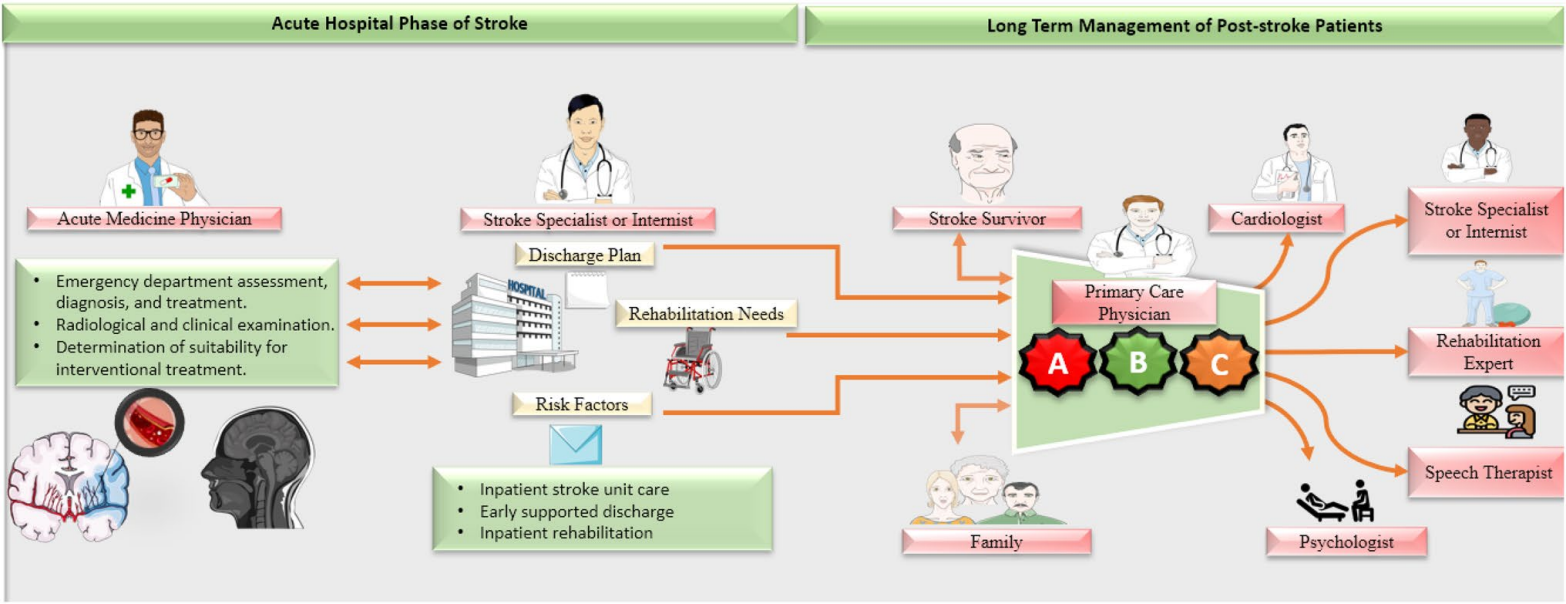
Collaboration of multidisciplinary team with general practitioners at the centre. *A*  appropriate antithrombotic therapy, *B* Better functional and psychological status, *C* *c*omorbidities and lifestyle, patient values, and preferences Servier Medical Art images were used for this figure (https://smart.servier.com)

Patients with ischaemic stroke usually suffer from other major cardiovascular events due to the increased prevalence of multiple cardiovascular risk factors [48]. Management of these risk factors (including diabetes, smoking cessation, hyperlipidaemia, and especially hypertension) is vital in secondary stroke prevention. To optimise chronic disease management and minimise future major cardiovascular adverse events, a collaboration of physicians in a joint multidisciplinary team is essential. Since, in many countries, GPs are the first point of contact with the healthcare system, they can play an important role in care coordination (Fig. 2).

### Management of diabetes mellitus

Several studies have shown that stroke patients with diabetes have poorer functional and rehabilitation outcomes increased mortality, and longer hospital stays [49]. About 30% of patients with acute ischaemic stroke have prediabetes or type 2 diabetes, which is related to an increase in the risk of stroke recurrence [50, 51]. In such patients, good glycaemic control improves outcome, with a glycosylated haemoglobin A1c (HbA1c) target of < 53 mmol/mol (7%) recommended [52]. Pioglitazone has been shown to reduce the risk of future stroke [52], but its adverse events especially related to bone fractures, weight gain and heart failure, have restricted its use [53]. Newer agents, such as glucagon-like peptide-1 (GLP-1) receptor agonists and sodium–glucose cotransporter 2 (SGLT-2) inhibitors, are helpful in patients with established cardiovascular disease reducing the risk of cardiovascular outcomes [54]. SGLT-2 inhibitors are especially beneficial in those with heart failure or chronic kidney disease. In addition to pharmacological treatment, exercise, maintaining a healthy weight, and having a healthy diet can prevent the progression of prediabetes to type 2 diabetes [55].

### Lipid modification

Lipid modification reduces cardiovascular events in stroke survivors. Current guidelines suggest intense statin therapy in patients after ischaemic stroke [8, 52]. In patients with established atherosclerotic cardiovascular disease, strict treatment targets a reduction of more than 50% in low-density cholesterol (LDL-C) and LDL-C < 1.4 mmol/L (< 55mg/dl) [37]. However, the Stroke Prevention by Aggressive Reduction in Cholesterol Levels (SPARCL) [43] and (Treat Stroke to Target) (TST) [56] trials point to an LDL-C target of < 70mg/dl in patients with non-cardioembolic stroke. Studies investigating the additive effect of ezetimibe or PCSK-9 inhibitors suggest that lower levels of LDL-C are safe and effective in reducing further cardiovascular outcomes [57, 58]. In most stroke patients, including those with AF (ESUS), intensive lipid-lowering should be pursued [59].

### Management of hypertension

High blood pressure (BP) is the strongest risk factor for stroke development, and it is a recurrence, so even a small degree of decrease in BP can reduce the risk of stroke [60]. BP tends to be elevated in the hyperacute stage of stroke, though it may slowly subside to a lower level after a few days. However, increased BP persists in 25%–30% of stroke survivors at their discharge from the hospital [61].

A systematic review and meta-analysis of 8 studies, including 33,774 patients with ischaemic stroke or transient ischaemic attacks, showed that, during a median of 25 months follow-up, subsequent stroke occurred in 7.9% of patients under BP-lowering treatments vs 9.7% in patients taking placebo. However, mortality was similar in both groups (7.3% and 7.9%, respectively) [62]. It seems that BP-lowering treatments may reduce the risk of cardiovascular death but do not affect the all-cause mortality risk. Although guidelines suggest a BP target of < 130/80mmHg, the target BP and choice of antihypertensive drugs should be tailored to the stroke aetiology and patient characteristics [8, 52].

### Lifestyle changes

Lifestyle changes, including a healthy diet and physical activity as basic components of the non-pharmacological approach, are essential to prevent recurrent strokes. In terms of healthy diets, recent guidelines recommend Mediterranean diets and low salt diets for stroke survivors. Some epidemiological diet studies showed that regular consumption of fish, and a high intake of fruits, vegetables, and fibre reduce the risk of stroke [63]. Similarly, low salt consumption and high potassium consumption are associated with lower stroke rates [64, 65]. On the contrary, high consumption of added fats and processed meats is associated with a 39% increase in stroke risk [66]. Choosing healthy meals can help reduce the risk of recurrent stroke.

When it comes to physical activity, because of the physical impairments, even simple tasks might be challenging for stroke survivors, and this results in being inclined to sit for lengthy periods. Therefore, it is vital to encourage stroke survivors to perform physical activities with support from physical and occupational therapists. Exercise training in stroke patients can improve hypertension, lipid profiles, glucose metabolism, insulin sensitivity, balance, gait speed, endurance, and disability [67]. Stroke survivors should engage in 40 min of moderate–vigorous-intensity aerobic activity three–four times per week when they can. Otherwise, physical activity should be tailored to their exercise tolerance, recovery stage, and other individual situations [8].

To summarise, in addition to a healthy diet and physical activity, smoking secession, low consumption of alcohol, and medication compliance are essential in patients with stroke. The vast majority of strokes can be prevented through BP control, a healthy diet, regular physical activity, and smoking cessation. Furthermore, Healthy lifestyle factors tend to cluster together so that those with regular physical exercise are likely to stop smoking, reduce alcohol and lose weight. Combinations of healthy lifestyle behaviours (such as regular exercise, and avoiding smoking and heavy drinking) have implications for reducing incidents of AF [68] and also, in case AF occurs, risks of AF-related complications [69, 70]. Also, targeting various risk factors has shown to have additive advantages for secondary stroke prevention; in particular, aspirin, statin and antihypertensive medications, combined with dietary modification and regular exercise, can result in an 80% cumulative risk reduction in recurrent vascular events [71]. All these lifestyle changes and management of comorbidities can be challenging for stroke survivors and their physicians. Although the benefits of a healthy lifestyle and vascular risk factor control are well documented, risk factors remain poorly controlled among stroke survivors. Thus, multidisciplinary support and coordination of subspecialties are needed instead of simple advice from their physician. At this point, GPs can manage the coordination of subspecialties to avoid polypharmacy and increase adherence, collaboration, and trust between patients and primary care doctors.

## The primary care perspective

Most stroke management guidelines and recommendations focus on the acute in-hospital phase, diagnosis, and treatment of stroke, focusing on increasing survival rates. Therefore, the initial effects of a stroke in the acute stage are well known. However, for many stroke survivors and their families, acute stroke is just the beginning of a long journey of living disability caused by stroke. Primary care physicians are essential in optimising chronic disease control and managing and minimising complications. Many stroke survivors may develop various complications within months or years following a stroke, and the primary care physician is in an ideal position to manage the prevention of these complications. Also, they may help their stroke patients by recognising the potential complications that might occur after a stroke. In terms of rehabilitation, comparing two post-stroke rehabilitation programmes in Spain (home-based rehabilitation and standard outpatient rehabilitation in a hospital setting) revealed that both groups exhibited statistically significant gains in each assessment after physical therapy [72]. Using functional scales, these improvements were better in home-based rehabilitation patients than in-hospital patients [72].

The absence of an integrated care approach may result in pitfalls during follow-up and neglect of crucial components of overall health, such as cognitive/emotional impairment in stroke patients [73]. Several studies investigated the adherence of primary care settings to secondary stroke prevention guidelines. They revealed that many of these patients were not optimally treated according to guidelines, potentially due to the lack of a holistic, multidisciplinary and patient-orientated follow-up (Table 1) [74–80]. Accordingly, a secondary cardiovascular prevention study including patients with previous stroke followed-up in primary care demonstrated that only 53% of them were treated with statins, and 59% did not achieve the optimal BP, despite using antihypertensive treatment [81]. Although there are recently published guidelines and an increase in the awareness of patients with ischaemic stroke in primary care, still less than 50% of patients treated in primary care settings reach the prespecified treatment goals, including BP and lipid management [77].

**Table 1:** Studies including ischemic stroke patients followed up in primary care settings

Study *Country*	Type	Year	n	Follow-up	Findings
Doogue et al. [74] *Ireland*	Cross sectional	2019	328	–	63.1% had BP controlled* 47.3% of those with BP ≥ 140/90 had adequate doses of all anti-hypertensives^#^
Han et al. [75] *Hong Kong*	Retrospective	2010 To 2018	466	8.7 years (median)	33.9% died 17.2% had recurrent stroke 12.2% developed CAD
Bansal et al. [80] *Singapore*	Retrospective Cohort study	2012–2016	19,529 (BP control) 14,714 (LDL control 9,046 (glycaemic control)	1 year	Optimal control^$^ - BP 71.4% - Diabetes 52.9% - Hyperlipidaemia 66.6%
Pedersen et al. [76, 77] *Norway*	Prospective	2011–2012	51	1 year	47% BP < 140/90 mmHg 27% LDL < 2mmol/L 10–11 GP consultations/year needed for follow-up of stable chronic conditions
Abdul Aziz et al. [78] *Malaysia*	Retrospective	2012	151	2.3 years (median)	71% BP ≤ 140/90 mmHg
de Weerd et al. [79] *Netherlands*	Cohort study	2006–2007	244	1 year	Most patients would like to see GP more regularly Measurements rates: - BP 84% - Glucose 28% - Cholesterol 40% Treatments often not given despite raised values ~ 25% offered lifestyle advice

^*^According to European Society of Hypertension/ European Society of Cardiology (ESH/ESC) and National Institute for Health and Care Excellence (NICE) guidelines

^#^According to the World Health Organisation-Defined Daily Dosing (WHO-DDD) recommendations

^$^American Heart Association/ American Stroke Association (AHA/ASA) Stroke 2014 guidelines

BP: Blood pressure, GP: General practitioner, CAD: Coronary artery disease

The management of stroke survivors is a complex, lengthy, and interdisciplinary process. During this process, GPs may encounter some challenges. For example, Australian guidelines recommend developing comprehensive discharge care plans with stroke survivors and caregivers and sharing them with GPs [82]. Still, nearly a third of Australian stroke survivors are discharged home without receiving a discharge care plan [83]. Additionally, communication of each individual’s stroke-related risk factors (such as falls, recurrent stroke, infection, and depression) and their rehabilitation needs are rarely shared with GPs. Therefore, it is still a challenge to translate the efficacy of the interventions reported in clinical trials into routine clinical practice. To achieve this, cooperation between stroke specialists, internists, and GPs is essential. Since stroke specialists and internists play a crucial role in the acute phase of stroke, they are well versed in patients’ medical backgrounds, risk factors and rehabilitation requirements. Therefore, a discharge plan developed by stroke specialists or internists, which identifies specific individual risk factors, and their rehabilitation needs, help GPs during the long-term management of post-stroke patients. On the other hand, GPs play a key role in re-referring stroke survivors for evaluation when necessary and supporting their rehabilitation in the community thus, timely communication between stroke specialists (or internists) and GPs is essential (including the provision of agreed-upon goals, plans for a return to work or employment, and information about their rehabilitation).

## Conclusion

Acute stroke is just the beginning of an extended challenge. After hospital discharge, many stroke survivors may develop a range of complications that necessitate the assistance of various specialities and coordination of subspecialties. However, they may feel abandoned due to a lack of an interdisciplinary and patient-centred approach. Family physicians can play an important role in the long-term care of stroke survivors by facilitating effective follow-up, information provision, and referral to specialist services when needed. Adequate, patient-centred, multidisciplinary support can help stroke survivors to help regain their independence or reduce dependence. For such work to succeed, close interdisciplinary collaboration between primary care physicians and other medical specialities is required. To bridge the gap between stroke specialists, family physicians and patients, an integrated care plan must provide a holistic and patient-oriented approach.
